# Computer-Assisted Mobile Phone Interviews in Low- and Middle-Income Countries Through a Total Survey Error Framework

**DOI:** 10.1093/poq/nfag023

**Published:** 2026-05-07

**Authors:** Abigail R Greenleaf, Huguette Diakabana, Charles Lau

**Affiliations:** Technical Specialist, ICAP at Columbia University, Mailman School of Public Health, Columbia University, New York, NY, US; and Assistant Professor, Department of Population and Family Health, Mailman School of Public Health, Columbia University, New York, NY, US; Independent Researcher, The Luminous Agency, Vevey, Switzerland; Deputy Global Research Director, Gallup, Washington, DC, US

## Abstract

Researchers increasingly use computer-assisted telephone interviewing (CATI) via mobile phones in low- and middle-income countries (LMIC). A nascent methodological literature explores representation and measurement error in these surveys, but knowledge is disparate, siloed across disciplines, countries, and research designs. Using the total survey error framework, this research synthesis summarizes findings from peer-reviewed methodological research on CATI in LMIC. We used a scoping review methodology to identify and review 38 peer-reviewed journal articles to answer two research questions: (1) Which study designs, topic areas, and total survey error components have been examined in CATI mobile phone surveys conducted in LMIC? and (2) What does the research say about representation and measurement errors in CATI mobile phone surveys in LMIC? Based on these findings, this research synthesis highlights when, where, and how CATI surveys can be used across LMIC.

## Background

The first nationally representative cross-national household surveys in low- and middle-income countries (LMIC)^1^ were the 1972–1984 World Fertility Surveys, followed by the International Labour Organization’s household income and expenditure surveys (1970s), the Living Standards Measurement Study and the Demographic and Health Surveys in the 1980s, and UNICEF’s Multiple Indicator Cluster Surveys in the 1990s, among others (Cleland and Verma 1989; Glauser 1992).

While survey modes in high-income countries (HIC) have evolved from face-to-face (FTF) to telephone (1980s) and web (2000s) surveys, LMIC have relied on FTF interviewing. However, over the past fifteen years, computer-assisted telephone interviewing via mobile phones (CATI) increased dramatically in LMIC, facilitated by improvements in telecommunication infrastructure and growth in mobile phone ownership (International Telecommunication Union [ITU] 2023). Following early adoption of CATI (e.g., Mahfoud et al. 2015; Dabalen et al. 2016; Larmarange et al. 2016), the COVID-19 pandemic rapidly accelerated CATI adoption because FTF surveys were not feasible, due to social distancing and lockdowns (Rahman et al. 2021; Glazerman et al. 2022). In addition to providing cost- and time-efficiency, CATI enables researchers to more easily collect data in conflict-affected areas such as Gaza (Stoddard et al. 2024), or vast, hard-to-reach areas such as Sudan (Kirui et al. 2024) and Myanmar (Lambrecht et al. 2023). In LMIC, CATI is used for myriad purposes, such as to provide real-time estimates of food insecurity (World Food Program 2024), to monitor the long-term impacts of the COVID-19 pandemic on socioeconomic well-being (World Bank 2022), and for policy and program evaluation (e.g., Setswe et al. 2015; Coffey, Spears, and Hathi 2020).

CATI’s long history in HIC (Lavrakas et al. 2017) is limited in its application to LMIC given that LMIC present very different contexts. For example, HIC phone surveys started in the 1970s with landlines, then added mobile phones, and subsequently diminished in use due to increased internet surveys. In contrast, LMIC skipped the landline phase, transitioning directly to mobile phones (World Economic Forum 2015). However, low mobile phone ownership in some LMIC often results in substantial coverage error. Furthermore, in LMIC, phone numbers are rarely geographically based, so there is limited auxiliary information appended to frames or samples; furthermore, phone number databases are rare. Whereas response rates are falling in HIC (Dutwin and Lavrakas 2016), the novelty and rarity of phone surveys results in higher response rates in LMIC. Greater linguistic diversity in LMIC means interviewers and respondents are less likely to share a native language (Lau et al. 2020). As a result, other than basic survey best practices, methodological lessons from HIC do not readily translate to LMIC—necessitating dedicated research tailored to LMIC.

A burgeoning literature has explored the methodology of LMIC CATI surveys but is disparate and siloed across disciplines, countries, and research designs. The review papers that exist generally focus on one discipline, and few focus on survey error (Gibson, Pariyo, et al. 2017; Greenleaf et al. 2017; Ouedraogo, Gaudart, and Dufour 2019; Gourlay et al. 2021; Arita et al. 2023). Given that we are in the nascent era of CATI in LMIC, synthesizing the methodological literature is critical to understand the constraints and benefits of CATI.

**Figure 1.**
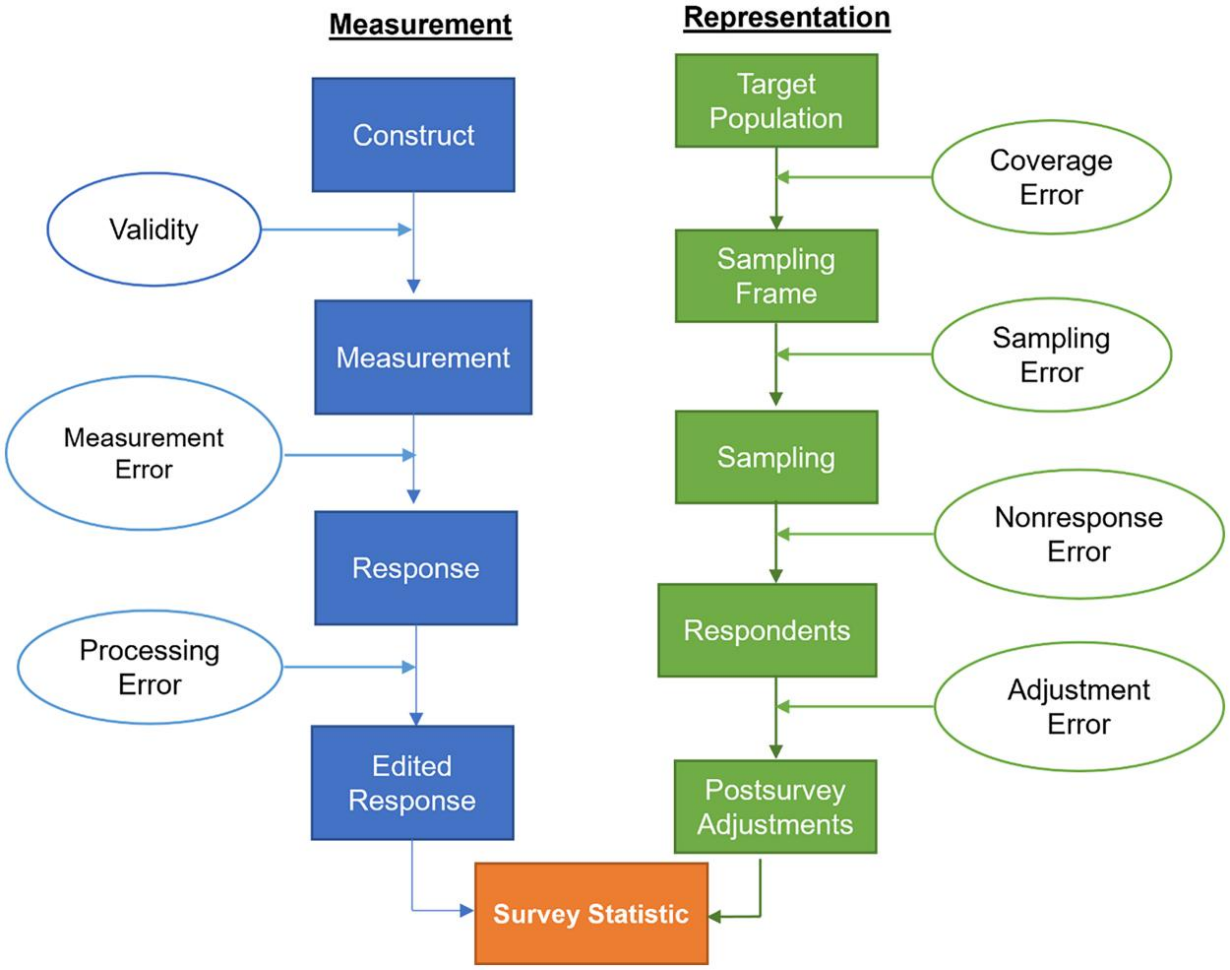
Total survey error. Reference for creation of figure: Groves and Lyberg (2010).

The objective of this research synthesis is to summarize findings about representation and measurement from peer-reviewed methodological research on CATI^2^ in LMIC. We anchor the research synthesis in the total survey error (TSE) theoretical framework (Groves and Lyberg 2010). The TSE framework provides a systematic approach to evaluate survey design and error, and also distinguishes between representation (who is included in the survey) and measurement (do answers reflect the constructs we are trying to measure), which helps pinpoint how error impacts survey estimates (see figure 1).

## Method

Because the goal of this research synthesis is to examine the extent and nature of survey design and error research among CATI surveys in LMIC, we used a scoping review methodology. This section explains how we applied the five-step framework for conducting a scoping review (Arksey and O’Malley 2005) and follows the Preferred Reporting Items for Systematic reviews and Meta-Analyses (PRISMA) Scoping Review Checklist (Tricco et al. 2018).

First, we drafted two research questions: “Which study designs, topic areas, and total survey error components have been examined in CATI mobile phone surveys conducted in LMIC?” and “What does the research say about representation and measurement errors in CATI mobile phone surveys in LMIC?”

Second, we drafted search terms and used seven search engines to cover a variety of subject matters. See table 1 for a list of the search engines and the search terms for PubMed, the search engine that returned the most results. All search engines were queried on June 4, 2024.

**Table 1.** Search engines utilized and search terms for PubMed.

Database	Search term
Agricultural & Environmental Science Collection	Basic + LMIC
EconLit	Basic
Embase	Basic + LMIC
Gender Studies Database	Basic
International Political Science Abstracts	Basic
PubMed	Basic + All countries
Web of Science	Basic + LMIC
Search terms Basic: ((“cell phone” AND survey) OR (“cell phone” AND questionnaire) OR (“mobile phone” AND survey) OR (“mobile phone” AND questionnaire) OR (CATI AND “data collection”) OR (“computer assisted telephone interview” AND survey) OR (“computer assisted telephone interview” AND questionnaire) OR (“computer assisted telephone interview” AND “data collection”)) LMIC: “low and middle income country” OR “low and middle income” All countries: Included all LMIC (*N* = 134) using the World Bank 2024 classification. For example: “afghanistan”[MeSH^a^ Terms] OR “afghanistan”[All Fields] OR “albania”[MeSH Terms] OR “albania”[All Fields] OR “algeria”[MeSH Terms] OR “algeria”[All Fields] OR “angola”[MeSH Terms] OR “angola”[All Fields]	

*Note:*  ^a^MeSH = medical subject heading.

Third, we selected studies of any subject matter based on our inclusion criteria, which were: (1) any target population other than a rare or highly specialized group, (2) a sample size greater than 100 (to avoid pilot or feasibility studies), (3) computer-assisted telephone interview (i.e., not interactive voice response or text message), (4) mobile-phone-based, (5) primary data collected for survey or surveillance^3^ (i.e., not a mobile-phone-based intervention such as call reminders to take medication, monitoring data, or an evaluation), (6) a total survey error component was included, (7) 2010–current (based on growth of mobile phone ownership and CATI), and (8) research conducted in an LMIC. Using these criteria, the two reviewers used Covidence software to screen titles and abstracts then review the full text. To ensure consistency of criteria and approach, authors ARG and CL applied the criteria together and discussed interpretations at the screening and extraction phase before working independently.

Fourth, following Pollock et al.’s (2023) recommendations, data was extracted into a Microsoft excel spreadsheet. Articles were screened, reviewed, and extracted by one reviewer unless there was an article that required a second opinion. The fifth and final step of collating, summarizing, and reporting results was conducted by the first and last author in an iterative process.

## Results

### Description of Studies

**Figure 2.**
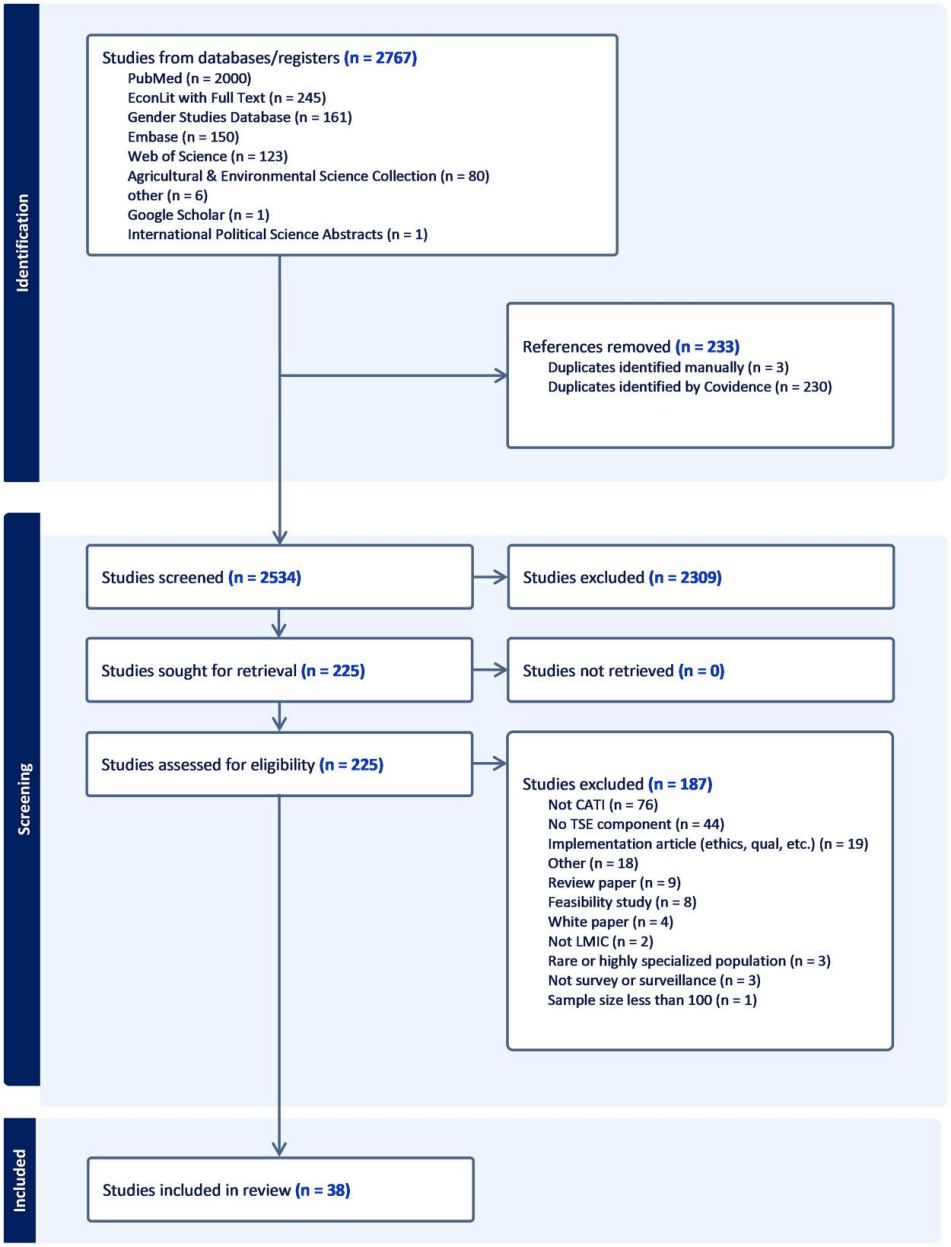
Flow diagram of manuscript selection process.

The seven-database search retrieved 2,761 records, and we contributed an additional six records. After 233 duplicates were removed, 2,534 titles and abstracts were screened, which resulted in 255 records that we reviewed in full. Using the above inclusion criteria, 38 studies were included in the review: 32 from databases, and six that were not retrieved during the search but which authors had knowledge of (see figure 2).

**Figure 3.**
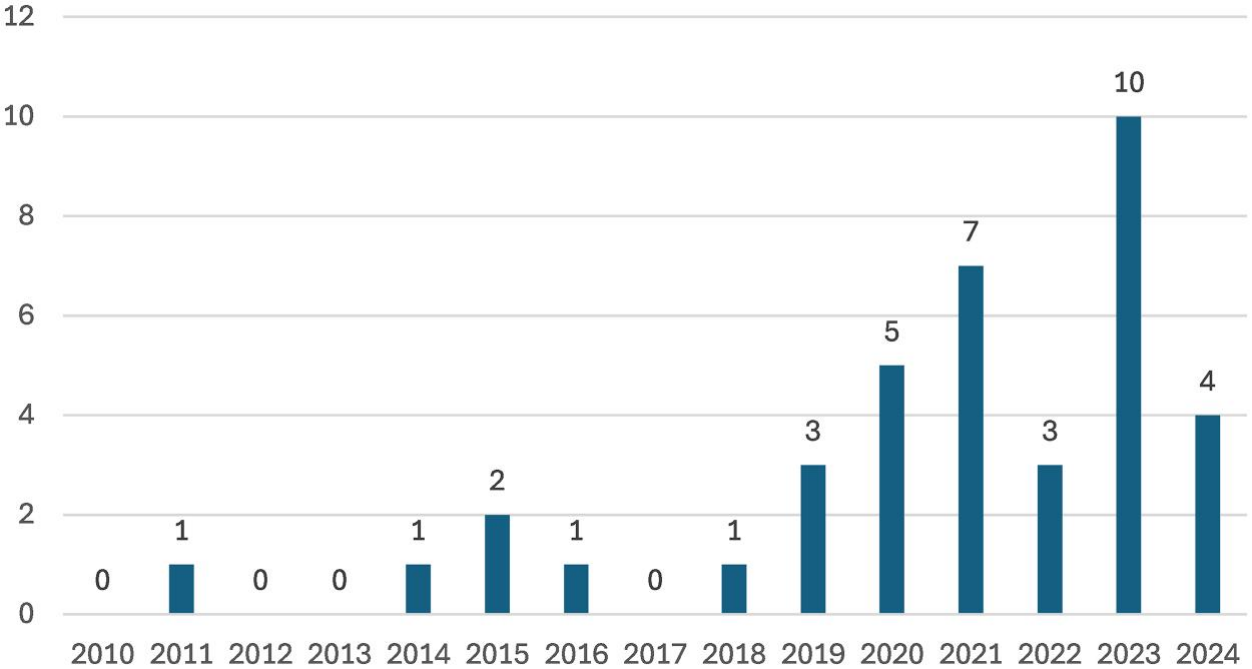
Number of LMIC CATI TSE publications from 2010 to 2024; 2024 reporting first half of the year only.

Of the 38 manuscripts included in this review, there were 36 unique studies across 21 LMIC.^4^ Three manuscripts reported on more than 1 country.^5^ There were 22 studies from Africa (East Africa = 8; West Africa = 8; Southern Africa = 5; North Africa = 1), 12 studies from South or Southeast Asia (India = 8; Bangladesh = 3; Myanmar = 1), 4 from Latin America, and 1 from the Middle East. The majority of studies focused on public health (*n* = 27), followed by socioeconomics (*n* = 5), agriculture (*n* = 2), and technology (*n* = 2). From 2020 to June 2024, 29 publications met our criteria (see figure 3). See Appendix table A1 for details on the studies.

Thirty studies were surveys, and six were public health surveillance. Most (*n* = 31) were cross-sectional, and six were longitudinal (one manuscript with multiple studies had both longitudinal and cross-sectional results). The most frequent and longest-duration longitudinal study was a COVID-19 phone surveillance system in Lesotho that called the same participants (with a sample refresh halfway through the study) weekly for 26 months (Lethoko et al. 2024). The majority of longitudinal studies called monthly or bimonthly, for 3–4 rounds.

Number of respondents ranged from 115 (Chen et al. 2021) to 154,494 (Gass et al. 2018). Of the 33 studies that reported the number of respondents, eight were studies with less than 1,000 participants, 12 with 1,000–2,000 participants, and 13 were greater than 2,000, of which two were over 10,000 respondents (Gass et al. 2018; Lambrecht et al. 2023). Among the 14 articles that reported length, interview length ranged from 5 to 45 minutes, with a median of 21.5 minutes.

Eleven studies reported that respondents received an incentive, of which nine reported the incentive amount. Of these nine studies, seven were cross-sectional and most (*n* = 5) provided $1 airtime, and the other two provided $1.37 and $1.50. For the longitudinal studies that reported incentive information, one study provided $0.50 per week, and the other $2.20 for the first two rounds, then increased to $3.50 for the last two rounds.

### Representation

From the TSE design perspective, representation is composed of the target population (set of units to be studied), sampling frame (units from the target population that have a chance to be selected into the survey sample), sample (the groups from which measurements will be sought), respondents (those successfully measured), and postsurvey adjustments (steps taken to improve the quality of survey estimates; Groves et al. 2009). In this section, we first describe the representation design and error elements from the articles we reviewed. Next, because few studies isolated the source of representation error, we describe overall representativeness of surveys by sampling method.

#### Target population

The most frequent target population was a general population (*n* = 26), of which most were conducted at the country (*n* = 17), state (*n* = 5), city (*n* = 3), and district level (*n* = 2). Women were the target population for eight studies, and healthcare workers for two studies.

#### Sampling frame

The most common design was random digit dialing (RDD, *n* = 16), in which numbers were randomly generated, typically using prefixes from the country’s mobile network operators. Phone numbers in most LMIC are not assigned geographically, so RDD frames cannot target sub-national geographies, with exceptions mainly in middle-income countries such as Mexico and Brazil. The second most common design was FTF follow-up (*n* = 13), where the frame consists of respondents who previously participated in an FTF survey who are subsequentially contacted by phone. In this research synthesis, we refer to this as an “FTF follow-up survey.” The time between the FTF survey and the phone survey ranged from 2 weeks to 19 months. Seven studies created the sample frame another way; see table 2 for details.

**Table 2.** Sampling approaches other than random digit dialing or face-to-face follow-up.

Authors	Target population; geography	Description of “other” sampling approach
Gignoux et al. 2020	General pop; state: Cameroon	Local community health workers previously collected all household phone numbers in the selected villages.
Lambrecht et al. 2023	General pop; country: Myanmar	Vendor database: The database was established through a combination of random digit dialing and adults who consented to be contacted for future participation in phone survey data collection.
Wilson et al. 2015	Women; city: Sudan	Women selected by chiefs from a master list of residents within the unit.
Gass et al. 2018	Women; state: India	Women who presented for childbirth at study facilities were approached for enrollment prior to their discharge through a verbal informed consent process.
Ramesh et al. 2023	General pop; district: India	Household sampling frame derived from the previous annual census.
Chen et al. 2021	Health workers; district: Mali	The program implementers provided the sampling frame, including all community health workers from the six program districts who had received training on the study topic and were actively providing services in their communities.
Nagpal et al. 2021	General pop; state: India	Previous cell phone survey.

#### Coverage error

Under-coverage (elements in the target population that do not, or cannot, appear in the sample frame) is of greatest concern due to variable levels of mobile phone ownership in LMIC. For example, ownership in 2024 was 66 percent in Malawi, 69 percent in Nigeria, 79 percent in Uganda, and 87 percent in Lesotho (Afrobarometer 2025). Across geographies, respondents who own a phone are more likely to be “males, urban residents, literate, married, and relatively wealthy” (Elkasabi and Khan 2023) compared to those without phones. A growing number of articles compare phone owners with nonowners but were not included in our study, as they did not meet inclusion criteria (Jadhav and Weis 2020; Olamoyegun et al. 2020; Doyle et al. 2021; Mistry et al. 2021; Al Kibria and Nayeem 2023).

To describe coverage error, those with and without cell phones should be compared. Three articles in our review addressed coverage error (Lamanna et al. 2019; Nagpal et al. 2021; Glazerman et al. 2023). Articles found that coverage error impacts the disenfranchised (e.g., the poor, women), with varying impacts on the outcome of interest.

#### Sampling

Of the 16 RDD studies, seven set quota targets based on recent Census or similar data to ensure that harder-to-reach groups were included in the sample. Age, gender, and location (urban/rural, or geographic region) were the most common quota variables. The nine RDD articles conducted without quotas used a variety of study designs. Of note, two studies (Brazil and Mexico) conducted multistage RDD by stratifying by state (using prefix) then sampling within each state (Moura et al. 2011; Gaitán-Rossi et al. 2021). Two studies sampled participants proportional to the market share of each mobile operator (Lau et al. 2019; Berry et al. 2021).

Among the 13 studies that were FTF follow-up, seven called back the full FTF frame and two called back half the frame (Brubaker, Kilic, and Wollburg 2021). Two studies followed the parent multistage sample design, sampling one participant per household (Greenleaf et al. 2023; Lethoko et al. 2024). One study called one participant per primary sampling unit (Shah et al. 2020), and one article did not sufficiently describe the sampling approach (Hersch et al. 2021).

We identified two sampling approaches explicitly designed to reduce coverage and nonresponse error. Four studies (two FTF follow-up, two RDD; each defined response outcomes differently) asked participants to pass the phone to either a female (Kenya—24 percent completion rate) (Glazerman et al. 2023) or opposite gender (India, 7–11 percent success rate) (Hersch et al. 2021), a nonphone owner in the household (25 percent response rate) (Greenleaf et al. 2023), or an adult age 60 or older (asked only those whose quota groups were filled, success rate unknown) (Al Kibria et al. 2024). The Glazerman et al. (2023) article finds that passing the phone improves representation of harder-to-reach groups (e.g., young women, lower wealth, rural residents), but does not eliminate outcome bias due to coverage, and referrals also increase cost. The Hersch et al. (2021) article notes that passing the phone to the opposite gender required multiple callbacks because men were more likely to pick up the phone than women. The second notable approach to reduce coverage error was asking the person who answered the phone call to provide a household listing, then randomly selecting a participant (Coffey et al. 2021), but the article did not evaluate the impact of this method.

#### Sampling error

Sampling errors arise from surveys measuring only a subset of the frame population. Some sampling error is expected with an interval of confidence. No article specifically addressed sampling error or bias. While outside of our review scope but an important contribution, Labrique et al. (2017) describes how, despite many persons in LMIC having more than one phone number, the probability of selecting a person twice is negligible.

#### Respondents

**Figure 4.**
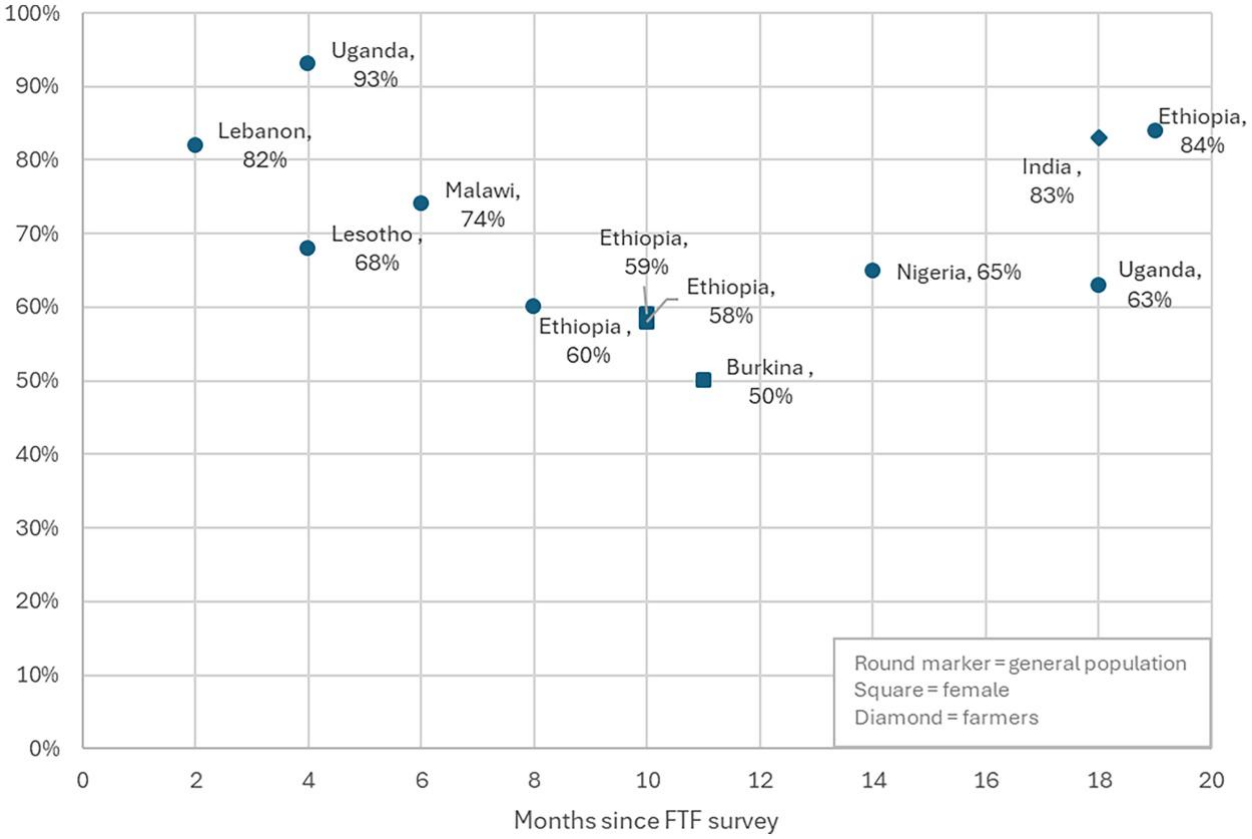
Response rates by months since collecting phone numbers. Only included response rate at first follow-up.

Response rates were inconsistently calculated, presenting challenges to drawing conclusions across studies. Nonetheless, of the 16 RDD studies, 12 reported a response or cooperation rate ranging from 3 percent to 52 percent. Among the 13 FTF follow-up studies, 10 reported a response rate. Response rates ranged from 50 percent to 93 percent, and follow-up time from two weeks to 19 months (see figure 4). The highest FTF follow-up response rate (93 percent) was among the general population in Uganda, with the CATI survey taking place in June 2020, which was both 4 months after the phone numbers were collected and during the COVID-19 pandemic (restricted movement) (Brubaker, Kilic, and Wollburg 2021). The lowest response rate was 50 percent, the rate among women of reproductive age in Burkina Faso contacted 8 months after an FTF survey (Greenleaf, Gadiaga, Choi, et al. 2020).

#### Nonresponse

Nonresponse arises when the values of statistics computed based only on respondent data differ from those based on the entire sample data (Groves and Lyberg 2010). While response rates are important for planning a CATI phone survey, a low response rate does not indicate response bias (Johnson and Wislar 2012).

Nonresponse is caused by noncontact (unreachable), refusal (uncooperative, including breakoff), or inability to participate (language, comprehension, etc.) (Massey and Tourangeau 2013). In nine articles, authors addressed causes of nonresponse. Noncontact was generally the largest source of nonresponse: For example, noncontact was 41 percent in Burkina Faso, which resulted in sample distortion (Greenleaf, Gadiaga, Choi, et al. 2020), 83 percent in Nigeria (Lau et al. 2019), 31 percent in India (Ramesh et al. 2023).

Eight studies examined cooperation (refusal and breakoff), of which two described differences in response rate by survey topic (mixed findings) (Coffey et al. 2021; Chasukwa et al. 2022), a third study addressed nonresponse by survey length (no error found) (Torrisi et al. 2024), and yet another examined response rates by key sociodemographics (no error found) (Lethoko et al. 2024). Several studies explored breakoff, and they found overall that breakoff was low, but differed by socio-demographics in Bangladesh (but not Tanzania) (Pariyo et al. 2023) and differed by survey topic (Plutowski and Zechmeister 2024), with lower breakoff rates when interesting survey content is earlier.

#### Postsurvey adjustment

Fourteen articles mentioned weighting, and two articles had extended descriptions of weighting (Brubaker, Kilic, and Wollburg 2021; Lambrecht et al. 2023). Poststratification was the most commonly used (*n* = 7) weight. Two studies, both based off FTF surveys with complex designs, used the FTF weights as the base weights then applied design, nonresponse, and poststratification weights (Greenleaf et al. 2023; Lethoko et al. 2024). Additional weighting methods include propensity score (Brubaker, Kilic, and Wollburg 2021), raking (Al Kibria, Ahmed, et al. 2023), weighting for multiple phone numbers (Larmarange et al. 2016), logit model with nonresponse (Abay et al. 2023), and calibration entropy (Lambrecht et al. 2023).

#### Adjustment error

Adjustment error occurs when the postsurvey adjustments meant to adjust for representation errors either do not improve the accuracy of or further distort the survey estimate. Nine articles addressed the effectiveness of the weights, often using a reference standard face-to-face survey to compare socio-demographic profile to create weights, and to compare subsequent outcomes.

Five studies found that the weights created a sample that was representative of other demographic factors not used in the construction of the weights (i.e., effectively matched the phone survey population with the target population). However, the weights had mixed results in improving the outcome of interest, meaning the weights could not fully address survey errors and bias. Two studies found comparability of key outcomes between CATI and FTF after weighting. In Myanmar, survey weights not only improved the representativeness of the weighted phone survey sample, but also key outcomes such as wealth and housing were comparable to the reference FTF survey (Lambrecht et al. 2023). The survey weights for an urban survey in Brazil were successful in matching the reference standard FTF survey sample characteristics as well as key outcomes (Moura et al. 2011). In Bangladesh, raking was effective at matching the demographics of the phone survey to a recent FTF survey, but the majority of study outcomes remained different between CATI and the reference FTF survey (Al Kibria, Ahmed, et al. 2023). Two studies did not compare the CATI and FTF outcomes of interest (Berry et al. 2021; Abay et al. 2023).

The four studies with unsuccessful weighting had different designs. Weighting the sample for multiple phone numbers for an RDD survey in Cote D’Ivoire improved (reduced) representation of men, but multiple notable differences between the weighted sample and target sample remained (Larmarange et al. 2016). Poststratification weights for an RDD female sample in Burkina Faso based on age, area of residence, and level of education were unsuccessful in moving the outcome of interest (modern contraceptive use) from 39 percent (weighted RDD) to the reference 26 percent in a concurrent FTF sample (Greenleaf, Gadiaga, Guiella, et al. 2020). Despite weights, a 2022 follow-up CATI survey in Uganda found that COVID-19 vaccination was much higher than the rate reported by the Ministry of Health at that time (Greenleaf et al. 2023). Finally, a multicountry study found that propensity score matching using household information to weight individual characteristics mitigated some but not all error (Brubaker, Kilic, and Wollburg 2021).

#### Overall representativeness of CATI surveys in LMIC

Above we described individual design and error elements of representation, but few articles decomposed the error sources. Therefore, we present overall representativeness—the compound effects of each error on representativeness—as described in twenty articles, by sampling method.

First, we summarize RDD designs. Middle-income countries created more representative samples than low-income countries. In low-income countries, RDD often produced systematic and larger representation errors. Table 3 summarizes underrepresentation for RDD and other sample designs, including illustrative examples that show magnitude of differences. Underrepresentation is most notable for less educated, asset-poor, and lower-income groups. Other groups that are often underrepresented include women, rural dwellers, and those from less populous regions.

**Table 3.** Patterns of underrepresentation in CATI surveys with RDD and other sample designs.

Type of underrepresentation	Article	Illustrative examples
Less education	Berry et al. 2021; Glazerman et al. 2023; Greenleaf, Gadiaga, Guiella, et al. 2020; Larmarange et al. 2016; Lau et al. 2019; Maffioli 2020; Pariyo et al. 2023; Ramesh et al. 2023	Côte d’Ivoire: 84% of the general population has primary or less education, compared to 48% in CATI (Larmarange et al. 2016). Nigeria: 65% of the general population has primary or less education compared to 14% in CATI (Lau et al. 2019).
Asset-poor	Glazerman et al. 2023; Maffioli 2020	Kenya (women only): In the FTF survey, durable floor, durable wall, and electricity were 52%, 51%, 42% respectively, compared to 58%, 71%, 87% in CATI (Glazerman et al. 2023). Liberia: Improved toilet is 37% in FTF survey and 87% in CATI (Maffioli et al. 2020).
Low-income	Nagpal et al. 2021; Ramesh et al. 2023	India: Poorest socioeconomic quintile was 20% in the census, but 16% in CATI (Ramesh et al. 2023).
Women	Larmarange et al. 2016; Lau et al. 2019; Maffioli 2020; Ramesh et al. 2023; Woelk et al. 2024	Bangladesh: 50% were female in census, compared to 37% in CATI (Pariyo et al. 2023). Tanzania: 51% were female in census, compared to 36% in CATI (Pariyo et al. 2023).
Rural dwellers	Larmarange et al. 2016; Lau et al. 2019; Maffioli 2020; Pariyo et al. 2023; Larmarange et al. 2016	Liberia: 60% are rural in FTF survey, compared to 30% in CATI (Maffioli 2020). Bangladesh: 77% were rural in Census, compared to 60% in CATI (Pariyo et al. 2023). Tanzania: 70% were rural in census, compared to 34% in CATI (Pariyo et al. 2023).
People living in less populous regions	Greenleaf, Gadiaga, Guiella, et al. 2020; Larmarange et al. 2016	Burkina Faso: 11% are in the Centre region (where capital is located), compared to 25% in CATI (unweighted) (Greenleaf, Gadiaga, Choi, et al. 2020). Côte d’Ivoire: 22% of the population lives in Abidjan (capital city) compared to 47% of CATI respondents (Larmarange et al. 2016).
Older adults	Glazerman et al. 2023; Greenleaf, Gadiaga, Guiella, et al. 2020; Larmarange et al. 2016; Lau et al. 2019; Pariyo et al. 2023	Nigeria: 50–64-year-olds comprise 17% of the general population, but only 7% of CATI respondents (Lau et al. 2019). Kenya (women only): Age 56+ was 15% in the FTF survey but 4% in CATI (Glazerman et al. 2023).

Next, we summarize FTF follow-up surveys, which are consistently more representative than RDD surveys. Four studies show that CATI surveys generally match, or have small deviations from, the sociodemographic characteristics from the FTF sampling frame: studies in Ethiopia (Abay et al. 2023), India (Anderson et al. 2024), Lesotho (Lethoko et al. 2024), and Uganda (Greenleaf et al. 2023). However, although a sample can be representative, the outcome of interest can still be biased, such as in Greenleaf et al. (2023). Other studies, however, showed errors in representation. For example, a CATI survey in Burkina Faso underrepresented younger ages (15–19), urban, lower wealth, and those without electricity (Greenleaf, Gadiaga, Choi, et al. 2020). Similarly, a four-country study (Ethiopia, Malawi, Nigeria, and Uganda) that examined individual-level characteristics found that CATI follow-up surveys underrepresented multiple groups (Brubaker, Kilic, and Wollburg 2021).

### Measurement

From a design perspective, measurement is organized by construct, measurement, response, and edited response. Measurement error pertains to deviations from answers given to a survey question and the underlying attribute being measured. The three types of measurement error are validity (the extent to which the measures reflect the underlying construct), measurement error (the observational gap between the ideal measurement and the response obtained), and processing error (the observational gap between the variable used in estimation and that provided by the respondent). No articles in our review addressed processing error, and only a few indirectly measured validity. The articles we reviewed focused largely on measurement error, which can stem from numerous sources (Biemer and Lyberg 2003).

Fifteen studies explicitly investigated measurement, of which 13 focused on factual questions about behaviors and events. The other two studies assessed respondent knowledge (Shah et al. 2020; Ng et al. 2022). In the remainder of this section, we describe how measurement error and bias is shaped by characteristics of questions, questionnaire length, respondents, and interviewers.

#### Question characteristics

Studies demonstrate a high level of measurement quality for questions that use binary responses (e.g., yes/no) to questions about simple constructs.

Two studies used repeated measure design and found high levels of measurement quality for questions using yes/no responses about straightforward noncommunicable disease risk factors such as cigarette smoking, alcohol consumption, diabetes, and other noncommunicable disease risk factors in Lebanon (Mahfoud et al. 2015), and in Bangladesh and Tanzania (Pariyo et al. 2019).

Three studies evaluated questions about food consumption and insecurity that used binary (yes/no) response options. In Mexico, eight items assessing household food insecurity (e.g., skipping breakfast, lunch, or dinner) produced reliable and valid data (Gaitán-Rossi et al. 2020), according to Rasch models (which evaluate internal psychometric validity) and tests of concurrent validity (which explored associations between food insecurity and anxiety). A study in Ethiopia found that data based on two separate series of questions (one that assessed *whether* a food item was consumed, and another assessing the number of days in the past 7 days) were consistent between separate CATI and FTF surveys (Abate et al. 2023). In Kenya, a series of yes/no questions about women’s consumption of ten different food groups produced similar data in both CATI and FTF modes (Lamanna et al. 2019).

Three studies demonstrated high levels of reliability and consistency for recall questions about binary outcomes, including a study about adverse birth outcomes in India (Gass et al. 2018), mortality and receipt of measles vaccine (Gignoux et al. 2020), and implementation of a community health program in Mali (Chen et al. 2021).

While data quality was good for simple questions, measurement quality is lower for four types of complex questions:

##### Hard-to-define constructs

Two studies find low levels of reliability for the questions about physical activity, a vague concept that requires lengthy definitions in the interview (Mahfoud et al. 2015; Pariyo et al. 2019).

##### Very detailed questions

In a study of community health workers in Mali, there were low levels of sensitivity for detailed questions (Chen et al. 2021).

##### Numeric questions

Two studies highlight significant challenges in collecting numeric or count data from respondents, with two phone surveys underestimating compared to the FTF, and one overestimating. First, respondent self-reports from CATI surveys about cookstove usage were double the cookstove sensor data (Wilson et al. 2015). Second, Anderson et al. (2023) found that the FTF survey produced significantly (14 to 68 percent less) lower crop production estimates compared to CATI, but ultimately both modes produced the same estimate of program impact despite the difference in specific estimates.

One study evaluated the quality of numeric data by comparing CATI and FTF surveys with respect to heaping. Heaping of agricultural outputs in India did not differ by mode (Anderson et al. 2023). In contrast, a review paper of six African countries (ineligible for this review) found that CATI surveys had significantly more age heaping than FTF surveys and censuses (Helleringer et al. 2023).

##### Knowledge questions

Two related studies estimated test-retest reliability of questions that assess knowledge about health in India. The populations were pregnant women (Ng et al. 2022) and community health workers (Shah et al. 2020). Among the former, overall knowledge was low, resulting in poor reliability for both modes. Among community health workers, reliability was low for complex questions (i.e., field-coded and count questions) but high for simple questions with yes/no responses.

#### Questionnaire length

Three studies explicitly studied the impact of questionnaire length on measurement quality, with mixed results. Torrisi et al. (2024) randomized respondents to receive a 10-, 20-, or 30-minute questionnaire in Malawi. Completion and cooperation rates were over 94 percent in all questionnaire versions. Indicators of data quality (item nonresponse, age heaping) did not vary by length, and the authors concluded that measurement error does not increase with length for their outcomes.

Abay et al. (2023) randomized the placement of a nutrition module within a CATI survey in Ethiopia. When nutrition questions are asked later in the interview (on average 15 minutes later), respondents reported a 28 percent decrease in minimum dietary diversity. The authors caution that fatigue may lead to underreporting later in the interview, especially because food recall is a cognitively demanding task. Third, when urban Ethiopians were asked about 118 types of food consumed in the past seven days and expenditure on 25 items, CATI produced 23 percent lower estimates of consumption than the FTF survey—doubling the poverty rate in CATI relative to FTF (Abate et al. 2023). An experiment in this study (randomizing the order of the 118 foods) suggests that survey fatigue may have led CATI (but not FTF) respondents to omit certain foods from their self-reports (an 11 percent decrease when asked about a food later in the survey). There was no difference by order in the FTF survey.

#### Respondent characteristics

Only one study shows how respondent characteristics affect measurement quality. Abate et al. (2023) demonstrate that the underestimation of consumption expenditures in CATI is greater in less educated (compared to their more educated counterparts) and larger households (difficulty recalling or knowing expenditures from all household members).

Two studies suggest that self-reports are more accurate than proxy reports. In Kenya, dietary diversity of the female respondent is reliable in CATI, but not when caregivers are asked to report about diet of children age 6 to 23 months (Lamanna et al. 2019). A study about adverse birth outcomes in India finds greater inconsistency about adverse birth outcomes when the report came from someone other than the mother or the mother’s husband (Gass et al. 2018).

#### Interviewer characteristics

Lamanna et al. (2019) report that over 50 percent of the variance in self-reports about nutrition in Kenya was at the interviewer level. When collecting data from caregivers about children’s nutritional intake, male interviewers recorded fewer foods in CATI (but not FTF) than female interviewers. In contrast, Abate et al. (2023) did not find any evidence of interviewer effects.

## Discussion

### Summary

We are witnessing a profound transformation in survey methodology in LMIC. Just a decade ago, CATI was rarely used. Today, CATI is an integral tool for collecting data, but the growth of CATI surveys has dramatically outpaced methodological research. Our research synthesis attempts to consolidate the growing knowledge base: CATI mobile phone surveys have been more often conducted using RDD (44 percent) than FTF follow-up (36 percent) or another sampling approach (19 percent). Most studies were surveys (83 percent), cross-sectional (86 percent), in Africa (61 percent), and public health content (75 percent). Within representation, postsurvey adjustment and nonresponse error articles were more frequent than sampling error (no articles) or coverage error articles. No articles addressed processing error, and few mentioned validity. Based on these findings, we address three implications.

#### Factors with the greatest influence on the accuracy of data for an LMIC CATI survey

A principal consideration when making survey design decisions is often *accuracy*—that is, the extent to which data are based on a representative sample and are free from measurement errors. Our research synthesis suggests that CATI surveys are most accurate in middle-income countries and when measuring straightforward constructs with simple response options. Our research synthesis highlights the influence of three factors on accuracy: country context, target population, and survey topic.

##### Country context

The literature provides insight into the types of countries in which CATI could produce representative samples. World Bank country economy classification appears to have some bearing: CATI RDD surveys are more representative in upper-middle-income countries (e.g., Colombia) than in lower-middle-income countries (e.g., Kenya, Nigeria) or low-income countries (e.g., Burkina Faso). Studies in lower-income settings have at times created acceptably representative samples using FTF follow-up designs—even in low-income contexts such as Ethiopia and Uganda (e.g., Brubaker, Kilic, and Wollburg 2021). Nevertheless, challenges to creating accurate estimates persist with either sampling approach, even when weights are applied.

The level of mobile phone ownership in a country influences accuracy (coverage error) and at times cannot be corrected through weights. Furthermore, high mobile penetration alone does not guarantee representativeness: CATI RDD surveys in countries with high mobile penetration such as Kenya and Nigeria still show substantial biases in representation. Unfortunately, coverage error will not quickly disappear, especially in sub-Saharan Africa, where growth rates of phone ownership have slowed (GSMA 2023). Additional structural and cultural factors such as linguistic fractionalization and public attitudes toward surveys can affect representativeness, but have limited empirical findings.

##### Target population

Much of the methodological literature focuses on national surveys, but given the aforementioned challenges, CATI could be more effective for nonnationally representative target populations. For example, professionals such as community health workers have been successfully contacted for monitoring, evaluation (Chen et al. 2021), or to assess knowledge (Shah et al. 2020). In addition, recruiting participants from a specific location such as a school or health facility can provide data on a group with a common exposure or experience. For instance, recruiting women from antenatal clinics to longitudinally track quality of care via CATI is filling a gap in maternal and newborn healthcare quality measurement (Arsenault et al. 2024).

##### Questionnaire design

Mirroring what we know from survey research in high-income contexts (Fowler and Cosenza 2012), the existing literature shows that questionnaires should ask straightforward questions about simple constructs to create quality measurements. Even more so than FTF surveys, CATI questionnaires should ask concise questions using simple response options (e.g., yes/no) about simple constructs that do not require significant explanation (e.g., defining “moderate physical activity”).

#### How to maximize accuracy, especially in contexts where CATI is less than optimal

We recognize that researchers will adopt CATI, even when CATI is less than optimal (e.g., substantial coverage error due to inadequate mobile phone ownership or complex subject matter). These researchers may compromise sample representativeness or measurement quality because they cannot afford FTF surveys, cannot access locations (e.g., conflict zones), or prioritize other factors such as timeliness (e.g., to allocate humanitarian food assistance) (World Food Programme 2024). Our research synthesis provides suggestions for these types of CATI surveys throughout the survey cycle.

In the design phase, researchers should consider who may be harder to reach and adjust accordingly by oversampling a group or using quotas, for example (e.g., Gaitán-Rossi et al. 2021). Next, pretesting methods were rarely mentioned but can identify and resolve issues with the questionnaire, which would result in higher data quality.

When implementing a CATI survey, researchers can maximize representation by using structured callbacks of nonrespondents, vary the times and day, and use incentives to increase representation among low-income respondents (Nagpal et al. 2021). After data are collected, weighting can be used to adjust the sociodemographic characteristics of participants to match the target population, but the researcher should not assume that the outcome of interest is reflective of the true value just because the sample characteristics match the target population characteristics.

When analyzing CATI data, researchers should consider how representation biases may impact their conclusions. For example, for a study on child vaccination: If education is associated with phone ownership and vaccination, vaccination rates would be upwardly biased in a CATI survey. Further, we recommend that researchers acknowledge limitations of CATI, by benchmarking CATI samples against other data sources and noting potential sources of bias.

#### Priorities for methodological research in CATI

##### Representation

While it is common to compare CATI results to a reference FTF survey, or to compare CATI and IVR survey modes, different sampling approaches (e.g., FTF follow-up, RDD, or another approach) are rarely compared for the same sample population. Our work includes only one example of comparison of RDD and FTF follow-up for the same sample population, with results reported in two different manuscripts. To isolate the impact of sampling approach and country context on findings, multiple sampling modes should be compared simultaneously. Research should study factors leading to nonresponse and experiment with methods to reduce nonresponse that have been explored for interactive voice response (automated calls) (Gibson, Pariyo, et al. 2017). Specifically, researchers should study the number and timing of call attempts, incentives, and how to engage non-phone owners in the sample. While the studies we reviewed rarely mentioned theory, methodological experiments should be based on theory (e.g., Leverage-Salience Theory, Gamification Theory, Influence Theory) (Dillman 2014) that can structure thinking and create generalizable knowledge beyond one country or study. Researchers should also transparently report case dispositions and response rates using AAPOR guidelines (American Association for Public Opinion Research 2023). When conducting this review, we observed numerous inconsistent ways researchers calculated response rates.

##### Measurement

Many studies in our review reflect findings from HIC (e.g., highly detailed questions can produce significant measurement error). However, there are ample reasons to anticipate that the levels and nature of measurement error may be different in CATI surveys in LMIC compared to HIC. Future research should probe deeper into how the different cultural, demographic, linguistic, and economic contexts in LMIC shape measurement error. For example, given lower levels of literacy and educational attainment, CATI respondents may be more vulnerable to fatigue effects. Cultural norms in LMIC prioritize politeness and agreeableness, which may lead to more acquiescence. Norms in many LMIC also place a premium on face-to-face communication, evidenced by face-to-face interviewers spending significant time building rapport with respondents. Without this rapport, respondents may be less willing to provide valid and reliable responses, especially in less democratic countries. Interviewer effects may also loom large in CATI surveys in LMIC, given the greater linguistic and cultural diversity within LMIC.

##### Bridging disciplinary and geographic diversity

In high-income countries, research about CATI was generated largely by survey methodologists from academia, survey firms, and the government. Research was shared in a focused set of venues (e.g., journals such as *Public Opinion Quarterly* and survey methodology conferences). In contrast, knowledge about CATI in LMIC is generated by a much broader range of disciplines such as economics, public health, and public opinion. The diversity of disciplines makes it harder to disseminate research findings because researchers attend different conferences and publish in different journals. We encourage researchers and practitioners to share knowledge widely, especially in free and accessible methods such as webinars and events in LMIC that are more accessible to researchers based in the global south.

The umbrella term “LMIC” encompasses upper-middle-income countries such as Argentina but also low-income countries such as Chad. Both geographic diversity and the paucity of cross-national studies makes it more challenging to consolidate knowledge and understand the contexts in which CATI is more effective. Studies with multiple countries and standardized questionnaires and sampling (e.g., Collins et al. 2023) can help eliminate confounding factors and build the evidence base about what types of countries can produce high-quality CATI data.

### Limitations

Each article was reviewed by a single reviewer, which may have introduced subjectivity and reduced the reliability of the review process. Another limitation is that some relevant articles may have been missed, as many studies do not explicitly list “mobile phone survey” in their keywords, making them difficult to identify. Furthermore, we only included English language articles. Postsurvey adjustment success was judged by study authors without empirical parameters for success, making it difficult to compare weighting success across studies. Additionally, we did not include gray literature in the review (although we cited some notable studies), which potentially omits valuable insights from nontraditional sources such as reports, theses, or government publications.

### Looking Ahead: The Future of CATI

CATI is a recent survey mode in LMIC: 75 percent of review articles were published in 2020 or later. Methodological research is still emerging, and researchers are continuing to explore how CATI fits in the ever-expanding research tool kit. In this sense, the current stage of LMIC CATI resembles web surveys in high-income countries in their nascent phase (late 1990s and early 2000s). During that era, researchers were grappling with similar challenges such as undercoverage, nonresponse, and measurement quality (Couper 2000) but have since developed understanding of web surveys’ strengths and weaknesses (Evans and Mathur 2018). This analogy reminds us that we are only just beginning to understand the strengths and weaknesses of CATI across diverse countries, research designs, and applications.

## Data Availability

Replication data and documentation are not available because of the permission policy of the original data collector. The editors have waived *POQ*’s replication policy for this manuscript. Please contact the corresponding author for more information.

## Footnotes

1 In this research synthesis, we adopt the World Bank’s definition of low- and middle-income countries. (World Bank 2024)

2 We limit our review to CATI, the primary mode of mobile phone data collection in LMIC. Other researchers have used other mobile phone modes, such as interactive voice response (IVR) and short message service (SMS, or text messaging).

3 Surveillance, in the context of public health, is the continuous, systematic collection and analysis of health-related data for public health action, whereas surveys are typically one-time or periodic data collection efforts designed to answer specific research questions or assess population characteristics.

4
Lethoko et al. 2024 and Francis et al. 2023 (Lesotho); Pariyo et al. 2023 and Pariyo et al. 2019 (Tanzania and Bangladesh).

5
Pariyo et al. 2023 and Pariyo et al. 2019 (Tanzania and Bangladesh); Brubaker, Kilic, and Wollburg 2021 (Malawi, Ethiopia, Nigeria, and Uganda).

## Appendix. Details of the 36 Unique Studies Across 21 LMIC

**Table A1.** Articles included in the review.

Citation	Country or countries	Population	Design	Subject matter
Abate et al. 2023	Ethiopia	General population	Other	Public health
Abay et al. 2023	Ethiopia	Women	FTF follow-up	Public health
Abay et al. 2023	Ethiopia	Women	FTF follow-up	Socioeconomics
Anderson et al. 2024	India	General population	FTF follow-up	Agriculture
Berry et al. 2021	Bangladesh	General population	RDD	Agriculture
Brubaker, Kilic, and Wollburg 2021	Ethiopia, Malawi, Nigeria, Uganda	General population	FTF follow-up	Socioeconomics
Chasukwa et al. 2022	Malawi	General population	RDD	Public health
Chen et al. 2021	Mali	Health workers	Other	Public health
Coffey, Spears, and Hathi 2020	India	General population	RDD	Public health
Francis et al. 2023	Lesotho	General population	FTF follow-up	Public Health
Gaitán-Rossi et al. 2021	Mexico	General population	RDD	Public health
Gass et al. 2018	India	Women	Other	Public health
Gignoux et al. 2020	Cameroon	General population	Other	Public health
Glazerman et al. 2023	Kenya	General population	RDD	Socioeconomics
Greenleaf et al. 2023	Uganda	General population	FTF follow-up	Public health
Greenleaf, Gadiaga, Guiella, et al. 2020	Burkina Faso	Women	RDD	Public health
Greenleaf, Gadiaga, Choi, et al. 2020	Burkina Faso	Women	FTF follow-up	Public health
Guzman-Tordecilla et al. 2023	Colombia	General population	RDD	Public health
Hersch et al. 2021	India	General population	FTF follow-up	Public health
Al Kibria, Ahmed, et al. 2023	Bangladesh	General population	RDD	Public health
Lamanna et al. 2019	Kenya	Women	FTF follow-up	Public health
Lambrecht et al. 2023	Myanmar	General population	Other	Socioeconomics
Larmarange et al. 2016	Cote d’Ivoire	General population	RDD	Public health
Lau et al. 2019	Nigeria	General population	RDD	Technology
Lethoko et al. 2024	Lesotho	General population	FTF follow-up	Public health
Maffioli 2020	Liberia	General population	RDD	Socioeconomics
Mahfoud et al. 2015	Lebanon	General population	FTF follow-up	Public health
Moura et al. 2011	Brazil	General population	RDD	Public health
Nagpal et al. 2021	India	General population	Other	Public health
Ng et al. 2022	India	Women	FTF follow-up	Public health
Pariyo et al. 2019	Bangladesh and Tanzania	General population	RDD	Public health
Pariyo et al. 2023	Bangladesh and Tanzania	General population	RDD	Public health
Plutowski and Zechmeister 2024	Haiti	General population	RDD	Public health
Ramesh et al. 2023	India	General population	Other	Public health
Shah et al. 2020	India	Health care workers	FTF follow-up	Public health
Torrisi et al. 2024	Malawi	General population	RDD	Public health
Wilson et al. 2015	Sudan	Women	Other	Technology
Woelk et al. 2024	Malawi	General population	RDD	Public health
